# Risk factors for immune-related adverse effects during CPI therapy in patients with head and neck malignancies – a single center study

**DOI:** 10.3389/fonc.2024.1287178

**Published:** 2024-02-14

**Authors:** Frederic Jungbauer, Annette Affolter, Christoph Brochhausen, Anne Lammert, Sonja Ludwig, Kirsten Merx, Nicole Rotter, Lena Huber

**Affiliations:** ^1^ Department of Otorhinolaryngology, Head- and Neck-Surgery, University Medical Centre Mannheim, Heidelberg University, Mannheim, Germany; ^2^ Department of Pathology, University Medical Centre Mannheim, Heidelberg University, Mannheim, Germany; ^3^ Department of Hematology and Oncology, University Medical Centre Mannheim, Heidelberg University, Mannheim, Germany

**Keywords:** HNSCC (head and neck squamous cell carcinoma), HNC (head and neck cancer), checkpoint inhibition, irAE, irAE diagnostic approach, PD-L1

## Abstract

**Introduction:**

Checkpoint inhibitors, such as PD1 inhibitors, represent an important pillar in the therapy of advanced malignancies of the head and neck region. The most relevant complications are immune-related adverse effects (irAEs), which represent an immense burden for patients. Currently, no sufficient stratification measures are available to identify patients at increased risk of irAEs. The aim of this retrospective study was to examine whether demographic, histopathological, clinical, or laboratory values at the start of CPI therapy represent a risk factor for the later occurrence of autoimmune complications.

**Material and methods:**

Data from 35 patients between 2018 and 2021 who received therapy with nivolumab or pembrolizumab for head and neck malignancy were analyzed and assessed for any associations with the subsequent occurrence of irAEs.

**Results:**

IrAE developed in 37% of patients, with pneumonitis being the most common form (14%). Pneumonitis was found in patients with an average significantly lower T-stage of primary tumors. An increase in basophilic leukocytes was found in patients with dermatitis later in the course. When thyroiditis developed later, the patients had a higher CPS score and lower monocyte levels.

**Discussion:**

Even though individual laboratory values at the beginning of therapy might show a statistical association with the later occurrence of irAEs, neither demographic, histopathological, nor laboratory chemistry values seem to be able to generate a sound and reliable risk profile for this type of complication. Therefore, patients need to be educated and sensitized to irAEs, and regular screening for irAEs should be carried out.

## Introduction

In the treatment of recurrent or metastasized, non-operable malignancies of the head and neck region (R/M-HNC), checkpoint inhibitors (CPI) represent an important therapeutic option. Human somatic cells are subject to immunosurveillance (1). Thus, not only potential external pathogens, such as bacteria or viruses, but also (pre)cancerous pathogens, are targeted by immune cells. Advanced tumors can also be infiltrated and attacked by immune cells, which has relevance in patient outcomes (2). The counter mechanism of tumor cells is called immunoevasion and comprises structures that individually and collectively ensure that tumor cells are either not recognized by the immune system or that antitumor immune responses are suppressed, which further promotes tumor progression. In doing so, tumor cells make use of regulatory mechanisms whose actual purpose is peripheral tolerance (i.e., they are intended to prevent autoimmunity through immunosuppressive activity) (3, 4).

An important pathway for immunoevasion is the programmed death 1 (PD-1) axis. PD-1 is a transmembrane glycoprotein consisting of 288 amino acids, which are expressed on the surfaces of T and B cells. It belongs to the immunoglobulin superfamily and inhibits the activity of T cells (5). PD-1 knockout mice develop autoimmune diseases, demonstrating the importance of PD-1 in regulating the immune system (6). Stimulation of PD-1 by its ligands PD-L1 and PD-L2 (7) triggers signaling cascades that prevent immune cells from targeting tumor cells (8). Based on this finding, new therapeutic approaches address the mechanism of immunoevasion (9).

The antibodies pembrolizumab and nivolumab bind to PD-1, thus blocking the PD-L1/PD-1 interaction, preventing the suppression of the immune cells. This enables the patient’s immune cells to detect the tumor cells and to act against them. Pretherapeutically, tumor tissue samples are used to determine the PD-L1 status, i.e., the expression of PD-L1 in the tumor tissue and the infiltrating immune cells. This serves to assess the probability of a successful treatment response. Nivolumab was first approved in Europe in 2015 for the treatment of malignant melanoma (10), and it was then approved for R/M-HNC in the U.S. in 2016 based on the results of the Checkmate 141 trial (11). Nivolumab is currently approved for R/M-HNC in adults with progression during or after platinum-based therapy. Pembrolizumab is approved as monotherapy or in combination with platinum and 5-fluorouracil (5-FU) chemotherapy for first-line treatment of R/M-HNC in adults with PD-L1-expressing tumors (combined positivity score “CPS” ≥ 1). Pembrolizumab is also approved as monotherapy for the treatment of R/M-HNC with PD-L1-expressing tumors (tumor proportion score “TPS” ≥ 50%) and cancer progression during or after prior platinum-based therapy in adults.

Typical side effects and complications of CPI are immune-related adverse events (irAEs). This occurs because the immune system, which is restored by CPI therapy, attacks not only the tumor cells but also the body’s own organs, as the natural self-tolerance mechanism is suppressed.

The most common irAEs include dermatological low-grade reactions, such as rash, pruritus, or vitiligo (12). Pulmonary inflammation, predominantly pneumonitis, is a potentially life-threatening irAE (13). Gastrointestinal inflammation, such as colitis and enterocolitis, also occurs and is primarily noted by diarrhea (14). Autoimmune hepatitis may also occur (15). irAEs affecting the endocrine system manifest most commonly as thyroiditis, pituitary inflammation, and much less commonly as diabetes mellitus (16). Cardiovascular irAEs also occur infrequently. They can manifest as myocarditis, pericarditis, or vasculitis, among others, and require careful monitoring because of their potentially fatal course (17, 18). Other irAEs involve the musculoskeletal system (19) and the nervous system (20). Some irAEs may be revealed by routine laboratory blood chemistry checks (especially those of the endocrine system); others require an inquiry by the patient about relevant symptoms (e.g., diarrhea). Depending on the severity of the irAEs, CPI therapy is continued, paused, or terminated, and immunosuppressive therapy, typically with cortisone, is initiated if necessary.

These irAEs represent a relevant burden for patients and pose a threat to effective antitumor therapy. However, the possibility of using biomarkers to assess the risk of the occurrence of these irAEs is very limited. Reviews of risk factors for irAEs on CPI therapy exist, but these do not include HNC patients due to a lack of data (21).

Therefore, the aim of this retrospective study was to examine whether demographic, histopathological, clinical, or laboratory values at the start of CPI therapy represent a risk factor for the later occurrence of autoimmune complications.

## Materials and methods

All patients who had received CPI therapy with pembrolizumab or nivolumab in the Department of Otolaryngology, Head- and Neck-Surgery of Mannheim University Hospital between January 1, 2018, and December 31, 2021, were included. Clinical, histopathological, and laboratory chemistry data were extracted from digital patient records (**Table 1**). Statistical analysis was performed using GNU PSPP 1.6.2 software. Descriptive analyses, t-tests, and bivariate correlations were performed. A p-value ≤ 0.05 was considered statistically significant. The results are given in absolute numbers ± standard deviations. The study was approved by the local ethics committee of Heidelberg University (# 2022-838).

**Table 1:** Excerpt from the clinical/histopathological data of the study collective: CPI (Checkpoint inhibitor therapy), irAE (immune related adverse event).

Patient No	sex [male; female]	age at start of CPI [years]	primary tumor	p16 [0=negativ, 1=positiv]	CPI drug [pembrolizumab; nivolumab]	irAE	T(umor) stage at time of first diagnosis	N(odal) stage at time of first diagnosis	M(etastasis) stage at time of first diagnosis	indication for CPI [1: local recurrence; 2: local residuum; 3: remote metastasis; 4: ADRISK study]	result first re-staging	previous chemotherapy	previous radiation treatment
1	m	82	oropharynx	1	pembrolizumab	dermatitis	1	0	0	3	favorable	no	yes
2	f	60	paranasal sinus	not examined	nivolumab	0	3	1	0	3	unfavorable	yes	yes
3	m	64	oropharynx	1	pembrolizumab	0	2	1	0	4	favorable	yes	yes
4	m	48	oropharynx	0	nivolumab	thyreoditis	3	3	0	1	favorable	yes	yes
5	m	62	oral cavity	1	pembrolizumab	arthritis	4	0	0	2	favorable	yes	yes
6	m	81	larynx	not examined	pembrolizumab	0	2	2	0	3	unfavorable	yes	yes
7	m	44	oropharynx	0	nivolumab	0	4	2	0	3	unfavorable	yes	yes
8	m	73	oropharynx	1	pembrolizumab	0	1	2	1	3	favorable	yes	yes
9	f	80	oral cavity	0	nivolumab	pneumonitis / colitis	2	0	0	1	favorable	no	yes
10	m	65	oropharynx	0	nivolumab	pneumonitis / colitis	2	1	0	3	favorable	yes	yes
11	m	59	oropharynx	1	pembrolizumab	colitis	2	2	0	1	favorable	yes	yes
12	m	66	hypopharynx	not examined	pembrolizumab	0	3	2	0	3	favorable	yes	yes
13	m	74	oropharynx	1	pembrolizumab	0	2	1	0	4	favorable	yes	yes
14	m	80	oropharynx	0	pembrolizumab	0	3	0	0	3	died before restaging	no	yes
15	m	68	oropharynx	not examined	pembrolizumab	pneumonitis	2	0	0	1	favorable	no	yes
16	m	64	oropharynx	1	nivolumab	dermatitis / thyreoditis	4	2	1	3	unfavorable	yes	yes
17	m	40	oropharynx	1	nivolumab	0	4	1	0	3	died before restaging	yes	yes
18	m	55	oropharynx	not examined	pembrolizumab	0	4	2	1	2	died before restaging	yes	yes
19	f	74	larynx	not examined	pembrolizumab	0	1	0	0	1	died before restaging	no	yes
20	m	63	hypopharynx	0	nivolumab	0	4	0	0	3	unfavorable	no	yes
21	f	67	oropharynx	0	pembrolizumab	0	4	2	0	3	favorable	yes	yes
22	m	91	external auditory canal	not examined	pembrolizumab	arthritis	2	0	0	1	favorable	no	yes
23	m	55	oropharynx	0	pembrolizumab	vasculitis / hepatitis	1	3	1	3	unfavorable	yes	yes
24	m	76	hypopharynx	0	pembrolizumab	0	4	3	1	3	favorable	no	no
25	m	61	larynx	not examined	pembrolizumab	0	3	3	0	4	favorable	yes	yes
26	m	73	oropharynx	1	pembrolizumab	0	4	2	0	3	died before restaging	yes	yes
27	m	60	oropharynx	0	pembrolizumab	arthritis	4	0	1	3	favorable	yes	yes
28	m	61	hypopharynx	0	pembrolizumab	0	1	2	0	3	favorable	yes	yes
29	f	72	oropharynx	0	nivolumab	0	4	0	0	1	favorable	yes	yes
30	m	55	oropharynx	1	pembrolizumab	0	2	1	0	4	favorable	yes	yes
31	m	71	oropharynx	0	nivolumab	0	4	0	0	3	favorable	yes	yes
32	m	65	carcinoma of unknown primary	0	nivolumab	pneumonitis	0	3	1	3	favorable	yes	yes
33	f	66	oropharynx	0	pembrolizumab	0	4	2	0	3	favorable	yes	yes
34	m	50	oropharynx	1	nivolumab	pneumonitis	2	2	0	3	favorable	yes	yes
35	m	67	parotid gland	1	nivolumab	0	3	2	0	3	favorable	yes	yes

## Results

Within the study period, 35 patients were treated with CPI therapy. A total of 29 patients were male (82.9%), and 6 were female (17.1%). The mean age at initiation of CPI therapy was 65 years (± 11.1 years, range 40–91 years). A total of 22 patients received therapy with pembrolizumab (53.7%), and 13 received therapy with nivolumab (31.7%).

The primary tumor was located in the oropharynx in 22 patients (62.9%), in the hypopharynx in 4 patients (11.4%), in the larynx in 3 patients (8.6%), and in the oral cavity in 2 patients (5.7%). In one patient each (2.9% each), the primary tumor was found in the paranasal sinuses, external auditory canal, parotid gland, and carcinoma of unknown primary (CUP).

Human papillomavirus (HPV) status was determined by immunohistochemical staining of the surrogate parameter p16 as part of the histopathological routine assessment. A total of 15 tumors were determined to be p16-negative (42.9%); 12 tumors were determined to be p16-positive (34.3%); and in 8 tumors, p16-status was not examined (22.9%).

The indications for therapy with CPI were remote metastasis after platinum therapy in 19 patients (54.3%), unresectable recurrences in 8 patients (22.9%), and local residual tumors after primary therapy in 3 patients (8.6%). 5 patients received pembrolizumab in the ADRISK trial (ClinicalTrials.gov identifier: NCT03480672) in combination with cisplatin (14.3%).

Almost all patients received radiotherapy beforehand (n = 34), and the majority also received platinum-containing chemotherapy before starting CPI therapy (n = 27). There were no significant differences in the occurrence of irAE depending on any previous radio(chemo)therapy.

**Figure 1:**
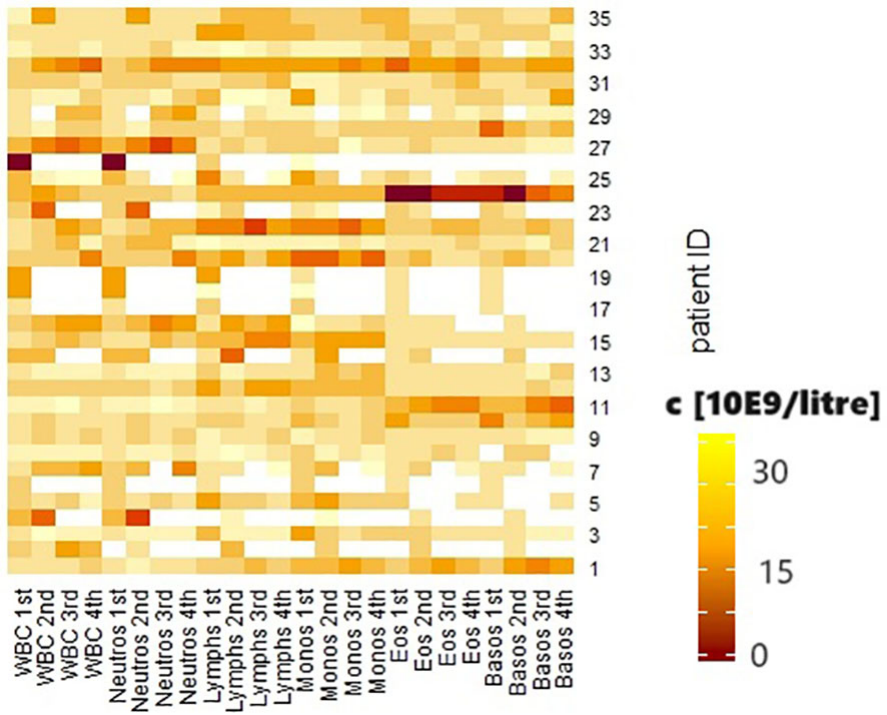
Heatmap of the biological values. White blood cells (WBC), neutrophil granulocytes (neutros), lymphocytes (lymphs), monocytes (monos), eosinophil granulocytes (eos), basophil granulocytes (basos) before the first, second, third and fourth courses of immunotherapy. Concentration shown in 10E9/liter.

Abuse of both alcohol and nicotine was present in 13 patients (37.1%); 12 patients used nicotine only (34.3%), and 3 patients used alcohol regularly (8.6%). Seven patients denied regular noxious substance use (20%). No statistically significant associations were shown between continued substance abuse and the occurrence of irAEs. Similarly, there were no statistically significant differences in the outcome of the first re-staging. Group comparisons between patients who did not consume noxious substances and those who consumed only nicotine showed a significantly higher relative proportion of lymphocytes in the differential blood count before the initiation of CPI therapy in patients without nicotine (p = 0.041). The group consuming only alcohol showed a significantly higher mean tumor status than the group without noxious substances (p = 0.004). This difference in tumor status was also seen in the comparison of the group without noxious substances with the patients who consumed both nicotine and alcohol (p = 0.028). Patients who used both noxious substances were significantly younger on average (72.43 ± 13.53 vs. 60.46 ± 9.8 years, p = 0.035). These patients also had a significantly lower proportion of p16-positive tumors (p = 0.018). Prior to initiation of CPI therapy, they showed higher leukocyte counts (p = 0.019), a significantly higher percentage of neutrophil granulocytes (p = 0.024), and a lower percentage of lymphocytes (p = 0.03) and basophil granulocytes (0 = 0.04) in the differential blood count. In the group comparison of patients who consumed at least one noxious substance and those who did not consume noxious substances, the first group showed a significantly higher T-stage (p = 0.003), a lower proportion of p16-positive tumors (p = 0.003), and higher relative proportions of neutrophil granulocytes (p = 0.05), and lower proportions of lymphocytes (p = 0.007) in the differential blood count at the beginning of therapy than the second group. The dynamics of the biological values are shown in **Figure 1**.

**Figure 2:**
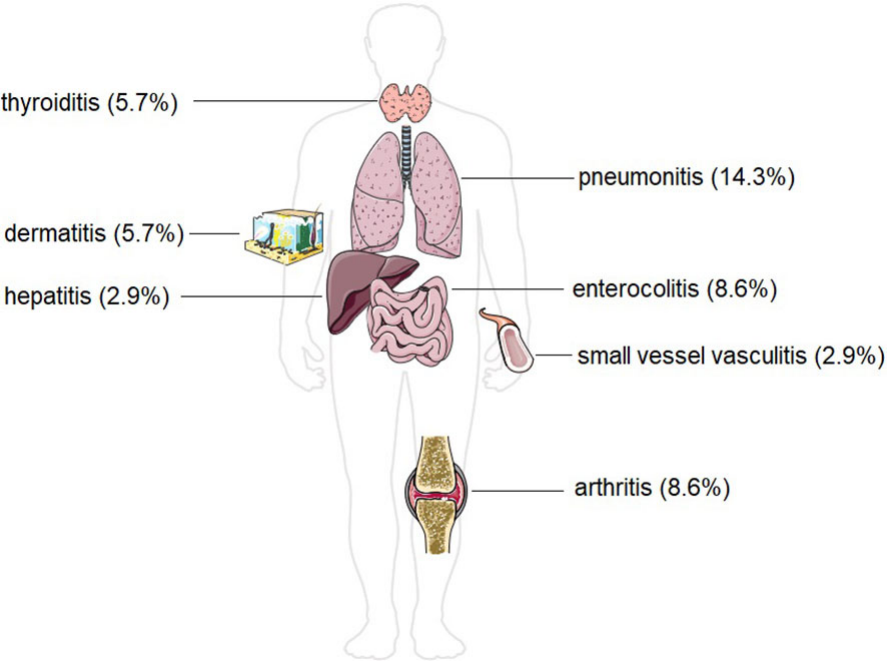
Incidence of different forms of immune-related adverse effects in our study group of patients with head and neck malignancies. The Figure was partly generated using Servier Medical Art, provided by Servier, licensed under a Creative Commons Attribution 3.0 unported license.

During the course of therapy, 13 patients experienced at least one irAE (37.1%) (**Figure 2**). Pneumonitis occurred in 5 patients (14.3%). Arthritis and enterocolitis occurred in 3 patients (8.6%) each. Dermatitis occurred in 2 patients (5.7%), and thyroiditis also occurred in 2 patients (5.7%). Small vessel vasculitis and autoimmune hepatitis occurred in 1 patient (2.9%).

Complications occurred at a mean of 15.3 weeks (± 16.6 weeks), with the earliest complication occurring after 2 weeks and the latest at 60 weeks after the first administration.

Under ongoing CPI treatment, dermatitis occurred on average after 12.5 weeks (± 3.5 weeks), thyroiditis after 6 weeks ( ± 5.6 weeks), arthritis after 3.3 weeks (± 0.5 weeks), pneumonitis after 26.2 weeks (± 21.3 weeks), and enterocolitis after 14.7 weeks (± 11.6 weeks). One patient developed vasculitis with concomitant hepatitis after 4 weeks.

The occurrence of dermatitis showed a significant association with the occurrence of thyroiditis in the same patient (p = 0.004). Patients with dermatitis during the study period had a significantly higher increase in relative basophil granulocytes between the second and third administrations (p = 0.005) and the third and fourth administrations (p = 0.001) of CPI compared to patients without dermatitis.

Patients who developed thyroiditis as an irAE showed significantly lower relative monocyte levels in the differential blood count at baseline (p = 0.029), which was also the case in the bloodwork done before the second administration of CPI (p = 0.007). In addition, they showed higher total leukocyte counts (p = 0.018) with higher relative (p = 0.045) and absolute (p = 0.007) neutrophil granulocytes. In addition, higher absolute values of basophilic granulocytes were seen at this time point (p = 0.044).

On average, patients with arthritis as an irAE showed significantly higher CPS scores (p = 0.006), lower nodal status (p = 0.022), higher differentiation of primary tumors (lower grading) (p = 0.021), and higher leukocyte levels (p = 0.048), with higher neutrophil granulocyte levels (p = 0.031) before the third administration of CPI than patients without arthritis.

The occurrence of pneumonitis was significantly associated with the delayed occurrence of enterocolitis (p = 0.006). Moreover, it occurred in patients with a significantly lower tumor size (p = 0.023).

The occurrence of enterocolitis was otherwise not significantly associated with any of the factors studied.

Because irAEs in the form of vasculitis and concomitant hepatitis occurred in only one patient in our collective, no meaningful statistical analysis of these two manifestations was possible.

Before the occurrence of any complications, two patients received oral cortisone therapy (5.7%) due to comorbidities (both patients received dexamethasone 4 mg, three times a day).

The occurrence of irAEs in general, as well as the individual subtypes, was not significantly associated with patient age or sex. There were no significant correlations between primary tumor localization and the occurrence of irAEs. There were no associations of the occurrence of irAEs in total in relation to any perineural sheath invasion (Pn), vein infiltration, lymphatic vessel infiltration (V, L), or p16 status. There was a significant association between smaller primary tumors and the occurrence of pneumonitis (p = 0.02, Somer’s D = -0.19), a low lymph node involvement (a smaller N stage), and the occurrence of arthritis (p = 0.05, Somer’s D = -0.16). A significant positive correlation was seen between lymph node status (N stage) and the possible presence of remote metastases (M status) (p = 0.027, r = 0.375). The negative correlation between N-stage and patient age was significant (p = 0.027, r = -0.374), as was the positive correlation between perineural sheath infiltration in the primary tumor and venous infiltration (p = 0.038, r = 0.427) and lymphatic vessel infiltration (p = 0.027, r = 0.450).

The presence of remote metastases correlated with a higher immune cell score (IC, percentage of area of PD-L1-positive immune cells from area of vital tumor cells) (p = 0.014, r = 0.484), while poorer tumor differentiation (corresponding to higher grading/G-value) correlated with a lower CPS value (p = 0.037, r = -0.419).

The ratio of neutrophil granulocytes to lymphocytes (NLR) showed no relevant correlations with the occurrence of irAEs or re-staging outcomes.

After the first re-staging, 13 patients showed a response in terms of partial remission (PR) (37.1%); 6 patients showed stable disease (SD) (17.1%); 6 patients showed progressive disease (PD) (17.1%); and 1 patient showed a mixed response (2.9%). The 4 patients who were treated in the ADRISK study showed a complete remission (CR) (11.4%). Five patients (14.3%) died before the first re-staging.

## Discussion

Compared to conventional chemotherapies, which for HNC are mainly represented by platinum-based drugs, the tolerability of CPI is rather good. Under cisplatin, typical complications occur, some of them lethal, such as renal failure (22), neuropathies (23), and myelosuppression with neutropenic susceptibility to infection and sepsis (24). In addition, patients typically suffer from marked nausea (25). The only targeted therapy in HNC is cetuximab, an antibody against the epidermal growth factor receptor, which is frequently administered and also generally well-tolerated (26).

IrAEs are the main complications of therapy with CPI. In our study, we recorded 37.1% of patients with these complications. It should be noted that, in some cases, the assignment of the symptoms that occurred (e.g., diarrhea or pneumonitis) or the laboratory chemical changes (e.g., increase in transaminases as an indication of hepatitis) cannot be reliably assigned to CPI therapy, as they can be due to multiple causes. In our study group, however, no other or more plausible causes were found in the subsequent clinical clarification. Moreover, the remission of symptoms or blood count changes after pausing CPI therapy and, if necessary, cortisone administration argued for their evaluation as irAEs. The most common irAE in our study was pneumonitis.

### Time of occurrence and progression

Both the frequency and the nature of irAEs vary depending on the tumor entity and CPI medication (27). It seems logical that organically manifested irAEs occur mainly in those anatomical areas where tumor cells are found, i.e., where activation of the disinhibited immune system occurs. Thus, pneumonitides are found mainly under CPI therapy in patients with non-small cell lung cancer (NSCLC) (28). However, the fact that the lung is also the primary metastatic site for HNC may explain why pulmonary irAEs are also relatively common in our patient population. Although not all of these patients had clinically/radiologically diagnosed pulmonary metastases, it seems possible that already scattered tumor cells in the lung provided a target for CPI and thus a preferential organ for irAE manifestation. This is consistent with other retrospective studies, in which pulmonary irAE was the most common in NSCLC and the second most common in HNC (29). Nevertheless, because pneumonitis also occurs as a relatively common irAE in other entities, an organotopically independent etiology also seems likely.

The time to onset of irAEs was highly variable in our study. While some patients developed irAEs after two weeks, i.e., after only one drug administration, in other patients they occurred only after a prolonged period of up to 60 weeks, i.e., after multiple drug administrations. The onset of arthritis after about three weeks seems to be the most stable in time; otherwise, the time of onset was widely scattered within the individual irAEs. The underlying pathomechanisms—why, in some patients, irAEs occur only after a very long delay—are still subject to research, as are the partly described chronic persistent irAEs, which did not occur in our cohort (30). Several different theories on the underlying pathomechanisms have been discussed thus far, on the basis of which the high heterogeneity of intensities and temporal occurrence of irAE could be explained. Most of them concern the individual nature of single components of the immune system, which are more or less predisposed to irAE. In this regard, a mismatch between T effector cells and regulatory T cells (Tregs) has been described. Tregs play an important role in peripheral tolerance, as demonstrated by mouse models, in which a deficiency of Tregs leads to a pronounced autoimmune response (31). Since PD-1 is expressed on Tregs, it can be assumed that CPIs used in the therapy of R/M-HNC also target Tregs in the tumor environment and lead to a shift in the balance between autoimmunity and tolerance (32). Thus, the occurrence of irAEs also depends on the initial presence and extent of the immunosuppressive effect of Tregs in an individual patient.

Histopathological studies have shown that infiltrates of specific T cells with similar T-cell receptor profiles were found in the tumors of patients, as well as in the organs affected by irAEs (33). Here, shared antigens in tumor tissue and endogenous healthy tissue seem to trigger the activation of specific autoimmune T-cell clones with consecutive inflammatory responses. Depending on the antigen distribution for these specific T cells in tumor tissue and patient organs, patients seem to be more or less susceptible to the development of irAEs. Furthermore, specific proinflammatory cytokine profiles seem to favor the development of irAEs (34). From this, Deng et al. deduced a more frequent occurrence of irAE in patients with a high body mass index, since overweight patients have a different cytokine profile, which apparently predisposes them to irAE more strongly (35). The role of preexisting autoantibodies prior to the initiation of CPI therapy is still controversial, and their significance also appears to be organ dependent. For instance, while researchers found a significant association of preexisting antithyroperoxidase antibodies and/or antithyrotropin receptor antibodies with the development of autoimmune thyroiditis (36, 37), patients with rheumatoid/arthritic irAEs showed few conventional rheumatoid autoantibodies, such as rheumatoid factor and anticyclic citrullinated peptide antibodies (38). In a study investigating the significance of autoantibodies during ipilimumab therapy, autoantibodies were associated with the occurrence of irAE but not necessarily with the respective organ-specific autoantibodies (39). Thus, there appears to be some underlying patient-specific profile that influences the likelihood of occurrence and the mode of irAE, but the significance of this observation cannot yet be conclusively quantified. Although much of the literature is devoted to the importance of T cells in the development of irAE, B cells also appear to have an influence, at least indirectly, on the development of irAE. Interestingly, in a study on the time course of irAE, a correlation was demonstrated between the decrease of B cells overall and the increase of a subset of CD21^lo^ B cells, as well as of plasmablasts (40).

In summary, the time course seems to be subject to multifactorial immunological, tumor-specific, and drug-specific influences and is currently unpredictable for individual patients.

### Demographic factors

The occurrence of irAEs in general, as well as the individual subtypes, was not significantly associated with patient age or sex. The literature of CPI therapies outside of HNC includes studies that attribute a risk factor for the occurrence of irAEs to young age (41) and sex (depending on the CPI, male or female) (42), as well as those that found no association with the occurrence of irAEs for either age (43) or sex (44). Overall, the likeliest summary is that the occurrence of irAEs in general, and the tolerability of CPI therapy in general, are not associated with age. However, specific subtypes, such as endocrine and gastrointestinal irAEs, seem to occur preferentially in younger patients, while skin and joint manifestations occur more often in older patients (43, 45). In this context, our data did not demonstrate an association with the occurrence of irAEs in general or their subtypes in HNC, despite a wide range of ages from 40 to 91 years in our cohort.

### Localization of the primary tumor

In head and neck oncology, depending on the localization of the primary tumor (oral cavity/pharynx/larynx, etc.), therapeutic approaches often vary; for example, depending on the localization, the resectability of a tumor is more or less feasible. It would have been conceivable that anatomic regions with increased lymphoepithelial tissue, such as the oropharynx, would have a stronger antitumor immune response, with a correspondingly higher risk of irAE. However, significant correlations between individual localizations and the occurrence of irAEs could not be demonstrated.

### Histopathological factors

Our results regarding the significant association of the occurrence of pneumonitis with a smaller tumor size and arthritis as an irAE with a lower N stage are controversial compared to previous studies on lung carcinoma and multiple melanoma patients (46). In those studies, T and N stages were not compared, but tumor burden in general was compared via the number of metastatic sites; a significant association was found between tumor burden and the occurrence of irAEs. It should be emphasized that the statistical association in our data was significant but rather weak (Somer’s D 0.16 and 0.19); appropriate caution should be exercised in evaluating this association. As a finding, however, it remains that insights from other entities regarding the association between tumor burden and the occurrence of irAEs cannot be readily applied to HNC patients.

No sufficient data to suggest histological tumor grade as a possible risk factor for the occurrence of irAEs are available.

### Dermatitis

Our HNC patients who developed dermatitis as an irAE during CPI therapy showed an increase in basophilic granulocytes over the course of the treatment period. Scientific evidence shows the role of basophil granulocytes in the development of allergic skin reactions (47) and inflammatory skin diseases (48). In addition, there are already studies describing an association between high basophil granulocytes and the occurrence of skin irAEs (49), albeit before the start of CPI therapy. In our data, a trend toward such correlations can also be recognized, derived, and statistically proven, but it is difficult to deduce a possible causal relationship. At present, no pathophysiological explanation for this has been found. Therefore, it is advisable to sensitize patients to the occurrence of skin changes and to make regular inquiries in this regard, as they are not necessarily associated with CPI therapy.

### Thyroiditis

Thyroiditis, as the most relevant representative of endocrinological irAEs, is mainly manifested by a new onset of hypothyroidism. Cases of initial hyperthyroidism with a subsequent transition to hypothyroidism have also been described in the literature (50), but this did not occur in our collective. Instead, all patients had transient asymptomatic hypothyroidism that regressed spontaneously during the course of treatment without further specific therapy.

When associated factors for the occurrence of thyroiditis as irAEs were examined, the main differences were seen in the differential blood counts of patients who developed thyroiditis and those who did not. Interpretation of the prognostic/predictive significance of leukocytes and their subsets is limited because of high variability, dynamics, and multiple influencing factors. Therefore, in previous studies, mainly histopathological examinations were performed, and tissue macrophages in the affected endocrine glands were shown to be partly responsible for the autoimmune response (51). However, the extent to which increased tissue macrophage content might be related to decreased peripheral blood monocyte content remains speculative. In contrast, when biomarkers for irAE were identified during CPI therapy in melanoma patients, the occurrence of pancreatitis was associated with elevated monocyte levels at therapy initiation (52). Thus, while different levels of monocytes appear to be related to the occurrence of endocrine irAEs, no clear direction or value can yet be demonstrated. Our data regarding elevated leukocyte levels with an increased proportion of neutrophil granulocytes are in line with previous observations in other entities, but there is limited valence, with a high variability of values (21).

### Arthritis

In other autoimmune rheumatoid diseases, such as adult-onset Still’s disease, elevated levels of leukocytes and neutrophil granulocytes are also typically found (53). Overall, this laboratory chemistry constellation is suggestive of a systemic inflammatory response and is not specific to irAE. The fact that these blood count changes occurred only before the third administration of CPI therapy compared to patients who did not develop arthritis does not support suitable usability as a biomarker for early detection. The high CPS levels found would be a possible explanation in the sense that, with strong PD-L1 expression, there is a greater target for CPI therapy and thus an increased risk for irAE. It must be emphasized that PD-L1 levels in the tumor do not necessarily represent those of the rest of the body’s cells. However, this association was found only in patients with arthritis as an irAE, not in the other irAE subgroups, so the predictive power of CPS seems questionable.

### Pneumonitis

Pulmonary irAE, especially pneumonitis, is a potentially life-threatening complication for patients and occurs mainly in those undergoing PD-1 inhibitor therapy (54). As discussed later, the predictive or prognostic significance of irAE remains the subject of current studies. However, the occurrence of pneumonitis during CPI therapy has already shown an association with worsened overall survival in NSCLC patients in studies (13). This illustrates that, for the evaluation of irAE, not only should the activated immune system be seen as a possible response to therapy, but the threat of the complication itself should also not be underestimated. The occurrence of pneumonitis under CPI therapy is difficult to predict. In a study of NSCLC patients receiving CPI therapy, the presence of high levels of baseline peripheral blood absolute eosinophil count was described as being associated with the development of pneumonitis irAE (55). Whether this also represents an independent risk factor could not be inferred by these authors. In contrast, no such laboratory associations were identified in our study. Other studies have found an association between the occurrence of pneumonitis and other metachronous irAE forms (29). It is possible that both NSCLC and HNC patients are susceptible to pulmonary diseases, such as irAE manifestations, because nicotine use is the most relevant risk factor for both entities. However, no statistical association between nicotine use and the occurrence of pneumonitis was found in our data, which is consistent with other studies that included HNC patients (29).

### Enterocolitis

Regarding enterocolitis as an irAE, our evaluations did not demonstrate any significant associations with the factors studied. In meta-analyses, the occurrence of enterocolitis as an irAE with CPI therapies is shown to be mainly dependent on the type of therapy (more frequent with CTLA-4 antibodies than with PD-1 antibodies) (56), while the localization and the entity of the tumor do not seem to influence the probability. It should be noted here that the symptoms of enterocolitis, such as diarrhea, also occur nonspecifically in the population, and even the clinical picture of pathological enterocolitis can be caused by multiple pathogens. Therefore, an etiologic assignment, such as irAE, is particularly difficult. The fact that the patients in our collective showed rapid clinical improvement after pausing CPI therapy and cortisone administration is not conclusive for an irAE, but from a retrospective perspective, it seems likely.

### Vasculitis and hepatitis

In our study, vasculitis and hepatitis occurred in only one patient. Therefore, a statistical evaluation of possible correlations and associations was not possible. Hepatic irAE manifests primarily in a laboratory-detectable elevation of the transaminases aspartate aminotransferase (AST) and alanine aminotransferase (ALT) (57). They occur more frequently in patients who are being treated with CPI due to hepatocellular carcinoma and who already have limited organ functions due to chronic hepatitis or liver cirrhosis (58). Patients are usually asymptomatic, and when symptoms do occur, they are often nonspecific and are therefore frequently not referred to therapy by patients (59). It is of high importance to regularly check liver enzymes during CPI therapy. As with other forms of irAE, other possible causes of liver enzyme elevation must be investigated and ruled out. These include infectious hepatitis and cholestasis.

Vasculitis as an irAE can occur in many forms due to its ubiquitous vascular supply. However, it is a rare subtype compared to the rest of the irAEs. In our patients, vasculitis manifestations were mainly found on the forearms and hands, with rapid regression under systemic cortisone therapy.

### Alcohol and nicotine consumption

There was no statistically significant association between the consumption of noxious substances by the patients and the occurrence of irAEs, nor was there any association with the outcome of the first re-staging. However, there were differences in the blood counts before the start of therapy, with increased leukocytes and neutrophil granulocytes and simultaneously lower levels of lymphocytes in the patients with toxicant consumption. Increased leukocytes, with a shift to increased formation of neutrophil granulocytes and decreased formation of lymphocytes, represent a systemic inflammation marker, as they can be increasingly detected with typical noxae, such as nicotine (60). Furthermore, patients with classical noxious consumption were found to have a greater tumor size at the onset of therapy. On one hand, it can be assumed that classical noxae (alcohol and tobacco) have a synergetic carcinogenic effect and thus explain the larger tumor sizes compared to abstinent patients. On the other hand, this could also be explained by the consideration that patients with increased use of noxious substances, on average, have rather low medical adherence and therefore consult a physician only at an advanced stage of the disease. The fact that the incidence of HPV-positive tumors seems to be lower in the subgroup with toxin use than in the subgroup without use is consistent with published studies in larger study cohorts (61).

### p16 status

In addition to classic noxae like alcohol and nicotine, infection with HPV, more specifically high-risk types 16 and 18, is a relevant risk and etiological factor in HNC (62). Therefore, histopathological determination of the expression of the surface marker p16 was performed in a large proportion of cases to assess whether HPV association of the tumor was present (63). However, it is important to emphasize that p16 is only a surrogate marker for HPV infection. Up to 23% of p16-overexpressing HNCs are not associated with HPV (64).

It seems conceivable that viral surface markers, which are also discussed as a cause for the better prognosis of HPV-positive vs. HPV-negative HNC (65), allow better recognition of tumor cells by immune cells infiltrating the tumor. Therefore, HPV- (or p16-) positive tumors could be expected to have a stronger immune response due to higher immunogenicity and are associated with both a higher risk for irAEs and a better outcome due to the stronger antitumor effect of CPI therapy. In our cohort, however, there was no significant difference between the p16-positive and p16-negative groups, either with regard to the frequency of irAEs or the outcome of the first re-staging. Early efficacy studies of pembrolizumab (66) and nivolumab (67) in R/M-HNC also showed no significant differences in outcome depending on HPV status. Recently, reviews and meta-analyses have demonstrated an improved outcome of p16-positive R/M-HNC under CPI. However, the study participants were treated with PD-1 inhibitors, as well as PD-L1 inhibitors, and no retrospective differentiation was possible (68). Some meta-analyses discovered that the superior outcome of p16-positive R/M-HNC was limited to oropharyngeal cancers only and was undetectable outside the oropharynx (69). This contrasts with other meta-analyses that also demonstrated the improved response of p16-positive R/M-HNC outside the oropharynx but without a distinction between PD-1 and PD-L1 inhibitors (70). It should be noted, however, that our study examined the outcome of the first re-staging, not the overall survival defined as an endpoint in most studies. By considering the first re-staging instead of overall survival as an endpoint, we hoped for a more specific assessment of the CPI response. Even though overall survival is considered one of the most relevant endpoints in oncologic research, it is unspecific in its assessment of treatment response because a variety of other factors are involved (e.g., comorbidities). Overall, the significance of HPV/p16 status as a prognostic parameter for CPI therapy in R/M-HNC does not appear to be as conclusively assessable as in conventional radio(chemo)therapy, where the more favorable outcome of p16-positive tumors is generally accepted (71).

### irAE as a predictive factor for re-staging

Among other antitumor drugs in the HNC field, namely the epidermal growth factor receptor (EGFR) antibody cetuximab, an association between the adverse drug effect in the sense of acne-like skin changes and the therapy response is known (72, 73). Thus far, it remains unclear whether a clinically visible autoimmune complication could also be a favorable predictive factor for treatment response, as it demonstrates a systemic activation of the immune system. In retrospective studies of HNC patients, the relationship between overall survival (OS) and progression-free survival (PFS) was investigated (74, 75). These authors describe the occurrence of autoimmune complications as an independent prognostic factor for favorable OS and PFS. Other studies and meta-analyses of this type confirm the association of autoimmune complications and later OS and PFS in other tumor entities, such as non-small cell lung cancer, melanoma, gastric cancer, renal cell carcinoma, and urothelial carcinoma (76). However, the authors criticize various sources of error, such as the guarantee (or immortal) time bias, which may feign this association due to the retrospective study design (77, 78). Responders to treatment with CPI survive longer, receive more CPI therapy, and thus have a cumulative increased risk of irAE over time. A retrospective evaluation demonstrated a spurious association between the occurrence of irAEs and a favorable outcome. To avoid this bias, we evaluated the treatment response at the first re-staging at a defined time point. Patients from our collective were re-staged after the first cycle (four administrations) of CPI using cross-sectional imaging (CT neck-thorax, possibly with CT abdomen in case of metastases), and the images were radiologically evaluated according to the RECIST criteria (79). Subsequently, patients were presented to our interdisciplinary head and neck tumor board, and a decision was made regarding the continuation of CPI therapy. In the case of PR, SD, and MR, CPI therapy was continued; the re-staging results were designated as “favorable” for this study. In patients with PD, the therapy regimen was changed; these re-staging results were designated as “unfavorable” for this study.

Statistical analysis showed a significant association between the occurrence of pneumonitis as an irAE and a favorable re-staging outcome (p = 0.038, Somer’s d = 0.24). For the remaining individual irAEs and the occurrence of irAEs in general, there were no statistically significant associations with subsequent re-staging outcomes. In contrast, previous meta-analyses that did not include HNC patients partially demonstrated an association between the occurrence of endocrine, dermatological, and low-grade irAEs and the better efficacy of CPI therapy (80). Meta-analyses have shown that the occurrence of high-grade irAEs is associated with a better response to therapy but with worse overall survival (81), which makes clear that the occurrence of irAEs should not only be interpreted as a positive predictive factor, if at all, but also as a potential threat to the patient. However, data regarding R/M-HNC are still too limited to draw valid conclusions about the association between irAE and the response to CPI therapy and patient outcomes.

### PD-L1 scores

A special interest in the selection of CPI therapy is given to the PD-L1 score, which indicates the expression of PD-L1 on tumor cells by specifying TPS (percentage of PD-L1-positive tumor cells from all vital tumor cells), IC (percentage of area of PD-L1-positive immune cells from area of vital tumor cells), and CPS (combination of TPS and IC, percentage of PD-L1-positive cells, including lymphocytes and macrophages from all vital tumor cells) quantified in the expression of PD-L1 on the tumor cells and the immune cells infiltrating them. There were no correlations between PD-L1 scores and the occurrence of irAEs. Similarly, there were no correlations between PD-L1 scores and the outcome of the first re-staging. Other studies have described a statistically significant association between tumors scored as “PD-L1-positive” and “PD-L1-negative” with respect to patient outcome (82), but correlation analyses showed no significant association once the PD-L1 score exceeded the positivity level (83). In the literature, studies of a possible predictive/prognostic value of PD-L1 scores beyond a positive/negative assessment can be found mainly in entities other than HNC, such as NSCLC (84). In patients receiving first-line CPI therapy, a positive correlation between high TPS scores and the occurrence of irAE could be demonstrated. In contrast, a correlation of high TPS scores and a favorable outcome was only found in patients who received CPI therapy as second- or third-line therapy, but not in first-line therapy. It should be noted that, depending on the study, the threshold for the evaluation of the PD-L1 score as positive or negative was chosen differently, which makes comparative considerations difficult. Overall, the determination of the PD-L1 score in the therapy of HNC is important for the assignment of the appropriate CPI agent, but beyond that, prognostic/predictive evaluation seems to be very limited.

### Concomitant cortisone therapy

In studies evaluating the efficacy of nivolumab in recurrent brain tumors against bevacizumab, a vascular endothelial growth factor (VEGF) inhibitor, the outcome of patients treated with cortisone in addition to nivolumab was found to be worse than in patients treated with nivolumab without cortisone (85). This effect was not found in the bevacizumab group, so it can be assumed that immunoinhibitory drugs, such as cortisone, limit the immunogenic antitumor effect of CPI. This seems comprehensible, as cortisone is also used for autoimmune complications with CPI therapy in HNC as a counter-regulation to disinhibit the immune system therapeutically (86). Similar results were also found in patients receiving CPI therapy for non-small cell lung cancer, with a worse outcome after high-dose cortisone therapy (87). However, the authors specifically note that the indication of cortisone therapy also reflects the advanced, more symptomatic tumor status of patients and therefore cannot necessarily be considered an independent factor. We reviewed the continuous medication of the patients in our study for any comedications with immunosuppressive agents they were receiving for other indications. Two patients were found to be on cortisone therapy for another indication prior to the possible occurrence of irAE. Both patients showed progressive diseases in the first re-staging but did not develop irAEs. This supports the idea that the antitumor effects of CPI therapy and potential irAEs are suppressed under continuous immunosuppressive medication. However, a potential statistical conclusion cannot be drawn, as the pattern described above comprises only a very small number of patients in our cohort (n = 2).

Furthermore, studies from other entities also exist in which no negative influence was demonstrated by immunosuppressive cortisone administration during CPI therapy. Nevertheless, these data refer to cortisone therapies initiated only during ongoing CPI therapy for the treatment of irAEs (i.e., in which an activated immune system is obviously present) (88, 89). In conclusion, it seems reasonable to check the patient’s existing permanent medication for potential immunosuppressants when starting CPI therapy and to critically evaluate the indications for this. At the same time, if irAEs occur that necessitate low-dose cortisone therapy, it should not be omitted out of false fear of the immunosuppressive effect on CPI therapy.

### Limitations

The limitations of this study are mainly the modest size of the study population. Since CPI is a relatively new treatment modality in HNC, clinical expertise is still limited compared to other entities in which CPI has been used for a longer time period. Furthermore, the retrospective evaluation cannot exclude influencing factors that were not documented. The patient population was also rather heterogeneous. On one hand, patients who are in recurrent or residual situations receive CPI therapy and no longer have surgical therapy options due to their multimorbidity. These patients are likely to succumb to their illness at an earlier stage before autoimmune complications occur.

On the other hand, patients with remote metastases and a likely poorer outcome who receive CPI over a long period of time show stable diseases throughout. Thus, the observation periods differed between the two groups due to the different dropout rates, which also resulted in limited comparability of the patient cases in the cohort. In addition, the guarantee-time bias mentioned above should not be neglected. It can also be assumed that irAEs with mild symptoms were not considered relevant by the patients and were not recorded, even despite specific inquiries at each examination. Thus, a certain underestimation of the occurrence of irAEs in general can be assumed, but not of clinically relevant irAEs.

## Conclusion

Our data show how difficult it is to predict autoimmune complications under CPI therapy. We were able to identify some parameters associated with the occurrence of irAEs in the retrospective evaluation. However, neither demographic, histopathological, nor laboratory chemistry values seem to be able to generate a sound and reliable risk profile for this type of complication.

There are only a few available studies on this topic regarding HNC patients, and few meta-analyses include HNC patients. While some of the data from our collective are consistent with findings from certain other tumor entities, others differ. Future multicenter studies must be designed prospectively to achieve robust data from ideally larger collectives.

Until then, it is all the more important to inform patients in detail about possible complications at the start of CPI therapy, to enquire regularly and specifically about corresponding symptoms, and to monitor organ functions by means of laboratory chemistry. By combining these precautions, autoimmune complications during CPI therapy can be detected at an early stage, and appropriate therapeutic measures can be initiated.

## Data availability statement

The original contributions presented in the study are included in the article/supplementary material. Further inquiries can be directed to the corresponding author/s.

## Ethics statement

The studies involving humans were approved by Ethikkommission II, Heidelberg university. The studies were conducted in accordance with the local legislation and institutional requirements. Written informed consent for participation was not required from the participants or the participants’ legal guardians/next of kin in accordance with the national legislation and institutional requirements.

## Author contributions

FJ: Conceptualization, Data curation, Investigation, Methodology, Writing – original draft. AA: Writing – review & editing. CB: Writing – review & editing. AL: Writing – review & editing. SL: Writing – review & editing. KM: Writing – review & editing. NR: Writing – review & editing. LH: Supervision, Writing – original draft.

## References

[1] Ferris RL. Immunology and immunotherapy of head and neck cancer. J Clin Oncol (2015) 33:3293–304. doi: 10.1200/JCO.2015.61.1509 PMC4586169 26351330

[2] Rajjoub S Basha SR Einhorn E Cohen MC Marvel DM Sewell DA. Prognostic significance of tumor-infiltrating lymphocytes in oropharyngeal cancer. Ear Nose Throat J (2007) 86:506–11. doi: 10.1177/014556130708600819 17915676

[3] Fife BT Pauken KE. The role of the PD-1 pathway in autoimmunity and peripheral tolerance. Ann N Y Acad Sci (2011) 1217:45–59. doi: 10.1111/j.1749-6632.2010.05919.x 21276005

[4] Seliger B Massa C Yang B Bethmann D Kappler M Eckert AW. Immune escape mechanisms and their clinical relevance in head and neck squamous cell carcinoma. Int J Mol Sci (2020) 21:3–6. doi: 10.3390/ijms21197032 PMC7582858 32987799

[5] Zhang X Schwartz JC Guo X Bhatia S Cao E Lorenz M. Structural and functional analysis of the costimulatory receptor programmed death-1. Immunity (2004) 20:337–47. doi: 10.1016/S1074-7613(04)00051-2 15030777

[6] Nishimura H Nose M Hiai H Minato N Honjo T. Development of lupus-like autoimmune diseases by disruption of the PD-1 gene encoding an ITIM motif-carrying immunoreceptor. Immunity (1999) 11:141–51. doi: 10.1016/S1074-7613(00)80089-8 10485649

[7] Latchman Y Wood CR Chernova T Chaudhary D Borde M Chernova I. PD-L2 is a second ligand for PD-1 and inhibits T cell activation. Nat Immunol (2001) 2:261–8. doi: 10.1038/85330 11224527

[8] Gianchecchi E Delfino DV Fierabracci A. Recent insights into the role of the PD-1/PD-L1 pathway in immunological tolerance and autoimmunity. Autoimmun Rev (2013) 12:1091–100. doi: 10.1016/j.autrev.2013.05.003 23792703

[9] Wu X Gu Z Chen Y Chen B Chen W Weng L. Application of PD-1 blockade in cancer immunotherapy. Comput Struct Biotechnol J (2019) 17:661–74. doi: 10.1016/j.csbj.2019.03.006 PMC6558092 31205619

[10] Zander H Muller-Egert S Zwiewka M Gross S Van Zandbergen G Engelbergs J. [Checkpoint inhibitors for cancer therapy]. Bundesgesundheitsblatt Gesundheitsforschung Gesundheitsschutz (2020) 63:1322–30. doi: 10.1007/s00103-020-03221-9 PMC7648013 33001218

[11] Ferris RL Blumenschein G Jr. Fayette J Guigay J Colevas AD Licitra L. Nivolumab for recurrent squamous-cell carcinoma of the head and neck. N Engl J Med (2016) 375:1856–67. doi: 10.1056/NEJMoa1602252 PMC5564292 27718784

[12] Belum VR Benhuri B Postow MA Hellmann MD Lesokhin AM Segal NH. Characterisation and management of dermatologic adverse events to agents targeting the PD-1 receptor. Eur J Cancer (2016) 60:12–25. doi: 10.1016/j.ejca.2016.02.010 27043866 PMC4998047

[13] Suresh K Psoter KJ Voong KR Shankar B Forde PM Ettinger DS. Impact of checkpoint inhibitor pneumonitis on survival in NSCLC patients receiving immune checkpoint immunotherapy. J Thorac Oncol (2019) 14:494–502. doi: 10.1016/j.jtho.2018.11.016 30503891

[14] Som A Mandaliya R Alsaadi D Farshidpour M Charabaty A Malhotra N. Immune checkpoint inhibitor-induced colitis: A comprehensive review. World J Clin cases (2019) 7:405–18. doi: 10.12998/wjcc.v7.i4.405 PMC6397821 30842952

[15] Spain L Diem S Larkin J. Management of toxicities of immune checkpoint inhibitors. Cancer Treat Rev (2016) 44:51–60. doi: 10.1016/j.ctrv.2016.02.001 26874776

[16] Naidoo J Page DB Li BT Connell LC Schindler K Lacouture ME. Toxicities of the anti-PD-1 and anti-PD-L1 immune checkpoint antibodies. Ann Oncol (2015) 26:2375–91. doi: 10.1093/annonc/mdv383 PMC6267867 26371282

[17] Salem JE Manouchehri A Moey M Lebrun-Vignes B Bastarache L Pariente A. Cardiovascular toxicities associated with immune checkpoint inhibitors: an observational, retrospective, pharmacovigilance study. Lancet Oncol (2018) 19:1579–89. doi: 10.1016/S1470-2045(18)30608-9 PMC6287923 30442497

[18] Wang DY Salem JE Cohen JV Chandra S Menzer C Ye F. Fatal toxic effects associated with immune checkpoint inhibitors: A systematic review and meta-analysis. JAMA Oncol (2018) 4:1721–8. doi: 10.1001/jamaoncol.2018.3923 PMC6440712 30242316

[19] Angelopoulou F Bogdanos D Dimitroulas T Sakkas L Daoussis D. Immune checkpoint inhibitor-induced musculoskeletal manifestations. Rheumatol Int (2021) 41:33–42. doi: 10.1007/s00296-020-04665-7 32743706

[20] Guidon AC Burton LB Chwalisz BK Hillis J Schaller TH Amato AA. Consensus disease definitions for neurologic immune-related adverse events of immune checkpoint inhibitors. J Immunother Cancer (2021) 9. doi: 10.1136/jitc-2021-002890corr1 PMC8291304 34281989

[21] Chennamadhavuni A Abushahin L Jin N Presley CJ Manne A. Risk factors and biomarkers for immune-related adverse events: A practical guide to identifying high-risk patients and rechallenging immune checkpoint inhibitors. Front Immunol (2022) 13:779691. doi: 10.3389/fimmu.2022.779691 35558065 PMC9086893

[22] Dierckes SJ Ragsdale ME Macik MR Weddle KJ. Retrospective analysis of the incidence and severity of acute kidney injury (AKI) in patients with head and neck cancer receiving weekly cisplatin with radiotherapy (RAISe-AKI). J Oncol Pharm Pract (2021) 27:1923–8. doi: 10.1177/1078155220978454 33302822

[23] Meier C Goldhirsch A Hess C Bazarian J Greiner R Beer M. [Polyneuropathy after cisplatin treatment]. Dtsch Med Wochenschr (1985) 110:721–5. doi: 10.1055/s-2008-1068894 4039651

[24] Barabas K Milner R Lurie D Adin C. Cisplatin: a review of toxicities and therapeutic applications. Vet Comp Oncol (2008) 6:1–18. doi: 10.1111/j.1476-5829.2007.00142.x 19178659

[25] Marty M. Ondansetron in the prophylaxis of acute cisplatin-induced nausea and vomiting. Eur J Cancer Clin Oncol (1989) 25 Suppl 1:S41–45. 2533898

[26] Taberna M Oliva M Mesia R. Cetuximab-containing combinations in locally advanced and recurrent or metastatic head and neck squamous cell carcinoma. Front Oncol (2019) 9:383. doi: 10.3389/fonc.2019.00383 31165040 PMC6536039

[27] Khoja L Day D Wei-Wu Chen T Siu LL Hansen AR. Tumour- and class-specific patterns of immune-related adverse events of immune checkpoint inhibitors: a systematic review. Ann Oncol (2017) 28:2377–85. doi: 10.1093/annonc/mdx286 28945858

[28] Ma K Lu Y Jiang S Tang J Li X Zhang Y. The relative risk and incidence of immune checkpoint inhibitors related pneumonitis in patients with advanced cancer: A meta-analysis. Front Pharmacol (2018) 9:1430. doi: 10.3389/fphar.2018.01430 30618738 PMC6297260

[29] Nobashi TW Nishimoto Y Kawata Y Yutani H Nakamura M Tsuji Y. Clinical and radiological features of immune checkpoint inhibitor-related pneumonitis in lung cancer and non-lung cancers. Br J Radiol (2020) 93:20200409. doi: 10.1259/bjr.20200409 32783627 PMC8519648

[30] Ghisoni E Wicky A Bouchaab H Imbimbo M Delyon J Gautron Moura B. Late-onset and long-lasting immune-related adverse events from immune checkpoint-inhibitors: An overlooked aspect in immunotherapy. Eur J Cancer (2021) 149:153–64. doi: 10.1016/j.ejca.2021.03.010 33865201

[31] Tanaka A Sakaguchi S. Regulatory T cells in cancer immunotherapy. Cell Res (2017) 27:109–18. doi: 10.1038/cr.2016.151 PMC5223231 27995907

[32] Najafi M Farhood B Mortezaee K. Contribution of regulatory T cells to cancer: A review. J Cell Physiol (2019) 234:7983–93. doi: 10.1002/jcp.27553 30317612

[33] Berner F Bomze D Diem S Ali OH Fassler M Ring S. Association of checkpoint inhibitor-induced toxic effects with shared cancer and tissue antigens in non-small cell lung cancer. JAMA Oncol (2019) 5:1043–7. doi: 10.1001/jamaoncol.2019.0402 PMC6487908 31021392

[34] Lim SY Lee JH Gide TN Menzies AM Guminski A Carlino MS. Circulating cytokines predict immune-related toxicity in melanoma patients receiving anti-PD-1-based immunotherapy. Clin Cancer Res (2019) 25:1557–63. doi: 10.1158/1078-0432.CCR-18-2795 30409824

[35] Deng T Lyon CJ Bergin S Caligiuri MA Hsueh WA. Obesity, inflammation, and cancer. Annu Rev Pathol (2016) 11:421–49. doi: 10.1146/annurev-pathol-012615-044359 27193454

[36] Osorio JC Ni A Chaft JE Pollina R Kasler MK Stephens D. Antibody-mediated thyroid dysfunction during T-cell checkpoint blockade in patients with non-small-cell lung cancer. Ann Oncol (2017) 28:583–9. doi: 10.1093/annonc/mdw640 PMC5834017 27998967

[37] Kobayashi T Iwama S Yasuda Y Okada N Tsunekawa T Onoue T. Patients with antithyroid antibodies are prone to develop destructive thyroiditis by nivolumab: A prospective study. J Endocr Soc (2018) 2:241–51. doi: 10.1210/js.2017-00432 PMC5836529 29600292

[38] Cappelli LC Gutierrez AK Bingham CO 3rd Shah AA. Rheumatic and musculoskeletal immune-related adverse events due to immune checkpoint inhibitors: A systematic review of the literature. Arthritis Care Res (Hoboken) (2017) 69:1751–63. doi: 10.1002/acr.23177 PMC5478477 27998041

[39] De Moel EC Rozeman EA Kapiteijn EH Verdegaal EME Grummels A Bakker JA. Autoantibody development under treatment with immune-checkpoint inhibitors. Cancer Immunol Res (2019) 7:6–11. doi: 10.1158/2326-6066.CIR-18-0245 30425107

[40] Das R Bar N Ferreira M Newman AM Zhang L Bailur JK. Early B cell changes predict autoimmunity following combination immune checkpoint blockade. J Clin Invest (2018) 128:715–20. doi: 10.1172/JCI96798 PMC5785243 29309048

[41] Asada M Mikami T Niimura T Zamami Y Uesawa Y Chuma M. The risk factors associated with immune checkpoint inhibitor-related pneumonitis. Oncology (2021) 99:256–9. doi: 10.1159/000512633 33477139

[42] Triggianese P Novelli L Galdiero MR Chimenti MS Conigliaro P Perricone R. Immune checkpoint inhibitors-induced autoimmunity: The impact of gender. Autoimmun Rev (2020) 19:102590. doi: 10.1016/j.autrev.2020.102590 32561463

[43] Samani A Zhang S Spiers L Mohamed AA Merrick S Tippu Z. Impact of age on the toxicity of immune checkpoint inhibition. J Immunother Cancer (2020) 8:5–6. doi: 10.1136/jitc-2020-000871 PMC7545628 33033183

[44] Jing Y Zhang Y Wang J Li K Chen X Heng J. Association between sex and immune-related adverse events during immune checkpoint inhibitor therapy. J Natl Cancer Inst (2021) 113:1396–404. doi: 10.1093/jnci/djab035 33705549

[45] Betof AS Nipp RD Giobbie-Hurder A Johnpulle R Rubin K Rubinstein SM. Impact of age on outcomes with immunotherapy for patients with melanoma. Oncologist (2017) 22:963–71. doi: 10.1634/theoncologist.2016-0450 PMC5553960 28476944

[46] Manne A Mulekar MS Escobar DE Alsayed A Sharma G Prodduturvar P. Clinical and hematological predictors of high-grade immune-related adverse events associated with immune checkpoint inhibitors. J Clin Med Res (2021) 13:268–75. doi: 10.14740/jocmr4511 PMC8166288 34104278

[47] Dvorak HF Mihm MC Jr. Basophilic leukocytes in allergic contact dermatitis. J Exp Med (1972) 135:235–54. doi: 10.1084/jem.135.2.235 PMC2180522 5060290

[48] Ito Y Satoh T Takayama K Miyagishi C Walls AF Yokozeki H. Basophil recruitment and activation in inflammatory skin diseases. Allergy (2011) 66:1107–13. doi: 10.1111/j.1398-9995.2011.02570.x 21371044

[49] Osawa T Inoue S Umeda M Hasegawa T Makino T Hori A. [Predictors of nivolumab-induced skin reactions]. Gan To Kagaku Ryoho (2018) 45:1533–5. 30382069

[50] Luo J Martucci VL Quandt Z Groha S Murray MH Lovly CM. Immunotherapy-mediated thyroid dysfunction: genetic risk and impact on outcomes with PD-1 blockade in non-small cell lung cancer. Clin Cancer Res (2021) 27:5131–40. doi: 10.1158/1078-0432.CCR-21-0921 PMC8815444 34244291

[51] Mihic-Probst D Reinehr M Dettwiler S Kolm I Britschgi C Kudura K. The role of macrophages type 2 and T-regs in immune checkpoint inhibitor related adverse events. Immunobiology (2020) 225:152009. doi: 10.1016/j.imbio.2020.152009 32962812

[52] Wolffer M Battke F Schulze M Feldhahn M Flatz L Martus P. Biomarkers associated with immune-related adverse events under checkpoint inhibitors in metastatic melanoma. Cancers (Basel) (2022) 14:8. doi: 10.3390/cancers14020302 PMC8773840 35053465

[53] Gerfaud-Valentin M Jamilloux Y Iwaz J Seve P. Adult-onset still's disease. Autoimmun Rev (2014) 13:708–22. doi: 10.1016/j.autrev.2014.01.058 24657513

[54] Su Q Zhu EC Wu JB Li T Hou YL Wang DY. Risk of pneumonitis and pneumonia associated with immune checkpoint inhibitors for solid tumors: A systematic review and meta-analysis. Front Immunol (2019) 10:108. doi: 10.3389/fimmu.2019.00108 30778352 PMC6369169

[55] Chu X Zhao J Zhou J Zhou F Jiang T Jiang S. Association of baseline peripheral-blood eosinophil count with immune checkpoint inhibitor-related pneumonitis and clinical outcomes in patients with non-small cell lung cancer receiving immune checkpoint inhibitors. Lung Cancer (2020) 150:76–82. doi: 10.1016/j.lungcan.2020.08.015 33080551

[56] Wang DY Ye F Zhao S Johnson DB. Incidence of immune checkpoint inhibitor-related colitis in solid tumor patients: A systematic review and meta-analysis. Oncoimmunology (2017) 6:e1344805. doi: 10.1080/2162402X.2017.1344805 29123955 PMC5665065

[57] De Martin E Michot JM Rosmorduc O Guettier C Samuel D. Liver toxicity as a limiting factor to the increasing use of immune checkpoint inhibitors. JHEP Rep (2020) 2:100170. doi: 10.1016/j.jhepr.2020.100170 33205034 PMC7648167

[58] Brown ZJ Heinrich B Steinberg SM Yu SJ Greten TF. Safety in treatment of hepatocellular carcinoma with immune checkpoint inhibitors as compared to melanoma and non-small cell lung cancer. J Immunother Cancer (2017) 5:93. doi: 10.1186/s40425-017-0298-2 29157287 PMC5697069

[59] Riveiro-Barciela M Barreira-Diaz A Vidal-Gonzalez J Munoz-Couselo E Martinez-Valle F Viladomiu L. Immune-related hepatitis related to checkpoint inhibitors: Clinical and prognostic factors. Liver Int (2020) 40:1906–16. doi: 10.1111/liv.14489 32329119

[60] Gumus F Solak I Eryilmaz MA. The effects of smoking on neutrophil/lymphocyte, platelet/ /lymphocyte ratios. Bratisl Lek Listy (2018) 119:116–9. doi: 10.4149/BLL_2018_023 29455548

[61] D'souza G Kreimer AR Viscidi R Pawlita M Fakhry C Koch WM. Case-control study of human papillomavirus and oropharyngeal cancer. N Engl J Med (2007) 356:1944–56. doi: 10.1056/NEJMoa065497 17494927

[62] Chaturvedi AK Engels EA Anderson WF Gillison ML. Incidence trends for human papillomavirus-related and -unrelated oral squamous cell carcinomas in the United States. J Clin Oncol (2008) 26:612–9. doi: 10.1200/JCO.2007.14.1713 18235120

[63] Pathak A Singh M Agarwal A Amit S. Determination of p16 overexpression as an indicator of human papillomavirus infection in oral dysplasia and carcinoma. Indian J Dent Res (2017) 28:418–23. doi: 10.4103/ijdr.IJDR_79_15 28836534

[64] Jouhi L Hagstrom J Atula T Makitie A. Is p16 an adequate surrogate for human papillomavirus status determination? Curr Opin Otolaryngol Head Neck Surg (2017) 25:108–12. doi: 10.1097/MOO.0000000000000341 28141601

[65] Fakhry C Westra WH Li S Cmelak A Ridge JA Pinto H. Improved survival of patients with human papillomavirus-positive head and neck squamous cell carcinoma in a prospective clinical trial. J Natl Cancer Inst (2008) 100:261–9. doi: 10.1093/jnci/djn011 18270337

[66] Bauml J Seiwert TY Pfister DG Worden F Liu SV Gilbert J. Pembrolizumab for platinum- and cetuximab-refractory head and neck cancer: results from a single-arm, phase II study. J Clin Oncol (2017) 35:1542–9. doi: 10.1200/JCO.2016.70.1524 PMC5946724 28328302

[67] Ferris RL Blumenschein G Jr. Fayette J Guigay J Colevas AD Licitra L. Nivolumab vs investigator's choice in recurrent or metastatic squamous cell carcinoma of the head and neck: 2-year long-term survival update of CheckMate 141 with analyses by tumor PD-L1 expression. Oral Oncol (2018) 81:45–51. doi: 10.1016/j.oraloncology.2018.04.008 29884413 PMC6563923

[68] Xu Y Zhu G Maroun CA Wu IXY Huang D Seiwert TY. Programmed death-1/programmed death-ligand 1-axis blockade in recurrent or metastatic head and neck squamous cell carcinoma stratified by human papillomavirus status: A systematic review and meta-analysis. Front Immunol (2021) 12:645170. doi: 10.3389/fimmu.2021.645170 33897693 PMC8058384

[69] Chakravarthy A Henderson S Thirdborough SM Ottensmeier CH Su X Lechner M. Human papillomavirus drives tumor development throughout the head and neck: improved prognosis is associated with an immune response largely restricted to the oropharynx. J Clin Oncol (2016) 34:4132–41. doi: 10.1200/JCO.2016.68.2955 PMC5477823 27863190

[70] Galvis MM Borges GA Oliveira TB Toledo IP Castilho RM Guerra ENS. Immunotherapy improves efficacy and safety of patients with HPV positive and negative head and neck cancer: A systematic review and meta-analysis. Crit Rev Oncol Hematol (2020) 150:102966. doi: 10.1016/j.critrevonc.2020.102966 32371338

[71] Lindel K Beer KT Laissue J Greiner RH Aebersold DM. Human papillomavirus positive squamous cell carcinoma of the oropharynx: a radiosensitive subgroup of head and neck carcinoma. Cancer (2001) 92:805–13. doi: 10.1002/1097-0142(20010815)92:4<805::AID-CNCR1386>3.0.CO;2-9 11550151

[72] Abdel-Rahman O Fouad M. Correlation of cetuximab-induced skin rash and outcomes of solid tumor patients treated with cetuximab: a systematic review and meta-analysis. Crit Rev Oncol Hematol (2015) 93:127–35. doi: 10.1016/j.critrevonc.2014.07.005 25139841

[73] Bar-Ad V Zhang QE Harari PM Axelrod R Rosenthal DI Trotti A. Correlation between the severity of cetuximab-induced skin rash and clinical outcome for head and neck cancer patients: the RTOG experience. Int J Radiat Oncol Biol Phys (2016) 95:1346–54. doi: 10.1016/j.ijrobp.2016.03.011 PMC5199017 27212198

[74] Economopoulou P Kotsantis I Papaxoinis G Gavrielatou N Anastasiou M Pantazopoulos A. Association of autoimmunity with survival in patients with recurrent/metastatic head and neck squamous cell carcinoma treated with nivolumab. Oral Oncol (2020) 111:105013. doi: 10.1016/j.oraloncology.2020.105013 32977184

[75] Foster CC Couey MA Kochanny SE Khattri A Acharya RK Tan YC. Immune-related adverse events are associated with improved response, progression-free survival, and overall survival for patients with head and neck cancer receiving immune checkpoint inhibitors. Cancer (2021) 127:4565–73. doi: 10.1002/cncr.33780 34547103

[76] Park R Lopes L Saeed A. Anti-PD-1/L1-associated immune-related adverse events as harbinger of favorable clinical outcome: systematic review and meta-analysis. Clin Transl Oncol (2021) 23:100–9. doi: 10.1007/s12094-020-02397-5 32495269

[77] Dall'olio FG Rizzo A Mollica V Massucci M Maggio I Massari F. Immortal time bias in the association between toxicity and response for immune checkpoint inhibitors: a meta-analysis. Immunotherapy (2021) 13:257–70. doi: 10.2217/imt-2020-0179 33225800

[78] Cheung KM Tsui TYM Chow JCH. Guarantee-time bias in studies on the relationship between immune-related adverse events and antitumor activity. Cancer (2022) 128:2549–50. doi: 10.1002/cncr.34244 35452127

[79] Eisenhauer EA Therasse P Bogaerts J Schwartz LH Sargent D Ford R. New response evaluation criteria in solid tumours: revised RECIST guideline (version 1.1). Eur J Cancer (2009) 45:228–47. doi: 10.1016/j.ejca.2008.10.026 19097774

[80] Zhou X Yao Z Yang H Liang N Zhang X Zhang F. Are immune-related adverse events associated with the efficacy of immune checkpoint inhibitors in patients with cancer? A systematic review and meta-analysis. BMC Med (2020) 18:87. doi: 10.1186/s12916-020-01549-2 32306958 PMC7169020

[81] Hussaini S Chehade R Boldt RG Raphael J Blanchette P Maleki Vareki S. Association between immune-related side effects and efficacy and benefit of immune checkpoint inhibitors - A systematic review and meta-analysis. Cancer Treat Rev (2021) 92:102134. doi: 10.1016/j.ctrv.2020.102134 33302134

[82] Ito T Okamoto I Tokashiki K Sato H Okada T Yamashita G. PD-L1 expression and survival rates using TPS and CPS for nivolumab-treated head-and-neck cancer. Anticancer Res (2022) 42:1547–54. doi: 10.21873/anticanres.15628 35220251

[83] Sanchez-Canteli M Granda-Diaz R Del Rio-Ibisate N Allonca E Lopez-Alvarez F Agorreta J. PD-L1 expression correlates with tumor-infiltrating lymphocytes and better prognosis in patients with HPV-negative head and neck squamous cell carcinomas. Cancer Immunol Immunother (2020) 69:2089–100. doi: 10.1007/s00262-020-02604-w PMC11027666 32448984

[84] Akazawa Y Yoshikawa A Kanazu M Yano Y Yamaguchi T Mori M. Non-small cell lung cancer with tumor proportion score > 90% could increase the risk of severe immune-related adverse events in first-line treatments with immune checkpoint inhibitors: A retrospective single-center study. Thorac Cancer (2022) 13:2450–8. doi: 10.1111/1759-7714.14576 PMC9436681 35820673

[85] Reardon DA Brandes AA Omuro A Mulholland P Lim M Wick A. Effect of nivolumab vs bevacizumab in patients with recurrent glioblastoma: the checkMate 143 phase 3 randomized clinical trial. JAMA Oncol (2020) 6:1003–10. doi: 10.1001/jamaoncol.2020.1024 PMC7243167 32437507

[86] Darnell EP Mooradian MJ Baruch EN Yilmaz M Reynolds KL. Immune-related adverse events (irAEs): diagnosis, management, and clinical pearls. Curr Oncol Rep (2020) 22:39. doi: 10.1007/s11912-020-0897-9 32200442

[87] Skribek M Rounis K Afshar S Grundberg O Friesland S Tsakonas G. Effect of corticosteroids on the outcome of patients with advanced non-small cell lung cancer treated with immune-checkpoint inhibitors. Eur J Cancer (2021) 145:245–54. doi: 10.1016/j.ejca.2020.12.012 33419647

[88] Horvat TZ Adel NG Dang TO Momtaz P Postow MA Callahan MK. Immune-related adverse events, need for systemic immunosuppression, and effects on survival and time to treatment failure in patients with melanoma treated with ipilimumab at memorial sloan kettering cancer center. J Clin Oncol (2015) 33:3193–8. doi: 10.1200/JCO.2015.60.8448 PMC5087335 26282644

[89] Fujii T Colen RR Bilen MA Hess KR Hajjar J Suarez-Almazor ME. Incidence of immune-related adverse events and its association with treatment outcomes: the MD Anderson Cancer Center experience. Invest New Drugs (2018) 36:638–46. doi: 10.1007/s10637-017-0534-0 PMC5962379 29159766

